# Intravitreal Administration of Human Bone Marrow CD34+ Stem Cells in a Murine Model of Retinal Degeneration

**DOI:** 10.1167/iovs.16-19252

**Published:** 2016-08-15

**Authors:** Elad Moisseiev, Zeljka Smit-McBride, Sharon Oltjen, Pengfei Zhang, Robert J. Zawadzki, Monica Motta, Christopher J. Murphy, Whitney Cary, Geralyn Annett, Jan A. Nolta, Susanna S. Park

**Affiliations:** 1 Department of Ophthalmology & Vision Science University of California Davis Eye Center, Sacramento, California, United States; 2 Department of Ophthalmology, Tel Aviv Medical Center, Sackler School of Medicine, Tel Aviv University, Tel Aviv, Israel; 3 Vitreoretinal Research Laboratory, University of California Davis Department of Ophthalmology, University of California, Davis, California, United States; 4 University of California Davis Research Investments in the Sciences and Engineering (RISE) Eye-Pod Laboratory, Department of Cell Biology and Human Anatomy, University of California, Davis, California, United States; 5 Department of Surgical and Radiological Sciences, School of Veterinary Medicine, University of California, Davis, California, United States; 6 Stem Cell Program, Institute for Regenerative Cures, University of California Davis Medical Center, Sacramento, California, United States

**Keywords:** CD34^+^, stem cells, intravitreal, retinal degeneration

## Abstract

**Purpose:**

Intravitreal murine lineage-negative bone marrow (BM) hematopoietic cells slow down retinal degeneration. Because human BM CD34+ hematopoietic cells are not precisely comparable to murine cells, this study examined the effect of intravitreal human BM CD34+ cells on the degenerating retina using a murine model.

**Methods:**

C3H/HeJ^rd1/rd1^ mice, immunosuppressed systemically with tacrolimus and rapamycin, were injected intravitreally with PBS (*n* = 16) or CD34+ cells (*n* = 16) isolated from human BM using a magnetic cell sorter and labeled with enhanced green fluorescent protein (EGFP). After 1 and 4 weeks, the injected eyes were imaged with scanning laser ophthalmoscopy (SLO)/optical coherence tomography (OCT) and tested with electroretinography (ERG). Eyes were harvested after euthanasia for immunohistochemical and microarray analysis of the retina.

**Results:**

In vivo SLO fundus imaging visualized EGFP-labeled cells within the eyes following intravitreal injection. Simultaneous OCT analysis localized the EGFP-labeled cells on the retinal surface resulting in a saw-toothed appearance. Immunohistochemical analysis of the retina identified EGFP-labeled cells on the retinal surface and adjacent to ganglion cells. Electroretinography testing showed a flat signal both at 1 and 4 weeks following injection in all eyes. Microarray analysis of the retina following cell injection showed altered expression of more than 300 mouse genes, predominantly those regulating photoreceptor function and maintenance and apoptosis.

**Conclusions:**

Intravitreal human BM CD34+ cells rapidly home to the degenerating retinal surface. Although a functional benefit of this cell therapy was not seen on ERG in this rapidly progressive retinal degeneration model, molecular changes in the retina associated with CD34+ cell therapy suggest potential trophic regenerative effects that warrant further exploration.

Retinal degenerative conditions, such as AMD and hereditary retinal degeneration, remain common causes of irreversible vision loss.^1^,^2^ Tissue regeneration is possible with stem cell therapy, and stem cells are being explored as a potential treatment for retinal degenerative disorders.^3^,^4^ Recent advances in stem cell research have made clinical studies possible in human patients with retinal degenerative disorders. Early phase clinical trials have been initiated using subretinal transplantation of fetal neural stem cells or retinal pigment epithelial cells derived from embryonic or induced pluripotent stem cells as treatment for hereditary or nonexudative AMD (www.clinicaltrials.gov; available in the public domain).^5^–^7^ These cell therapies appear to be well tolerated and may be capable of improving visual function. However, the use of these stem cells raises ethical and regulatory issues. In addition, allogeneic subretinal cell transplantation requires surgical manipulation of the central retina and prolonged systemic immunosuppression.

An alternative cell therapy approach that is being explored in clinical trial is intravitreal administration of autologous adult stem cells.^8^,^9^ This approach would be easier and potentially safer to administer because no systemic immunosuppression or retinal surgery would be needed. It has been shown in a murine model that intravitreal injection of autologous bone marrow (BM)-derived lineage negative hematopoietic stem cells can slow down hereditary retinal degeneration.^10^,^11^ One small phase 1 clinical study in Brazil treated patients with hereditary retinal degeneration with a single intravitreal administration of autologous mononuclear cells from the BM aspirate.^12^ The treatment was well tolerated, but visual benefit was minimal.

In human BM, hematopoietic stem cells are often characterized by the CD34+ cell surface marker. The mononuclear cell fraction of human bone marrow is a crude cellular mixture that contains a very low concentration of CD34+ cells (approximately 2%).^13^ Thus, by purifying the CD34+ cells from the BM aspirate and concentrating them, a potentially safer and more effective cell therapy might be delivered to the eye. Preclinical studies in eyes with acute ischemia-reperfusion injury or diabetic retinopathy demonstrated that these cells rapidly homed into the damaged retinal vasculature, where they appeared to play a role in tissue repair.^14^ No long-term ocular or systemic safety issues were noted with intravitreal administration of human BM CD34+ cells in NOD-SCID mice with acute ischemia-reperfusion injury.^15^ These human cells could be identified incorporated into the mouse retinal vasculature even 6 months following administration.

To date, there are no animal data investigating the effect of intravitreally administered human CD34+ cells from BM in eyes with retinal degeneration. However, a phase 1 clinical trial has been initiated exploring the effect of autologous intravitreal BM CD34+ cell therapy in patients with vision loss from retinal degeneration.^8^ No adverse effects were noted to be associated with the cell therapy during the 6-month study follow-up. Some eyes had improvement in visual function. Adaptive optics optical coherence tomography imaging showed new punctate hyperreflective changes within the degenerating retina suggestive of intraretinal incorporation of the cells injected into the vitreous cavity.^8^ However, no histologic correlate is available to correctly interpret these in vivo retinal imaging changes in human eyes.

The purpose of this current study is to further characterize the effect of the human CD34+ cells from BM on the degenerating retina following intravitreal administration using a murine model of hereditary retinal degeneration. By using an in vivo retinal imaging device designed for simultaneous multimodal imaging of murine eyes by scanning laser ophthalmoscopy (SLO) and optical coherence tomography (OCT), cellular changes within the retina potentially can be visualized in vivo following intravitreal injection of fluorescently labeled cells.^16^ By conducting molecular, functional, and histologic analysis of the degenerating retina following cell therapy, the potential therapeutic effect of these cells on the degenerating retina can be further investigated. The findings of this study would be important before exploring the potential therapeutic effect of intravitreal BM CD34+ cells in a larger clinical trial for patients with degenerative retinal conditions.

## Methods

### Animals

This study protocol was approved by the Institutional Animal Care and Use Committee at the University of California Davis before initiation. The study was conducted according to the approved protocol and in accordance with the ARVO statement for the Use of Animals in Ophthalmic and Vision Research.

Thirty-two C3H/HeJ mice homozygous for the rd1 mutation (C3H/HeJ^rd1/rd1^; Jackson Laboratories, Sacramento, CA, USA; strain 000659) were used in this study. All mice were male and 3 weeks of age at the initiation of the study and maintained at the Vivarium of the Institution for Regenerative Cures at the University of California Davis, which is a pathogen-free barrier facility that allows housing of immunosuppressed animals. Within 2–3 days following arrival to the vivarium, systemic immunosuppression was initiated in the mice by placement of the Alzet micro-osmotic pump (see section on Immunosuppression for details). Five days after initiating systemic immunosuppression, intravitreal injection of human CD34+ cells or saline (PBS) was performed only in the right eye under isoflurane anesthesia (see section on Intravitreal Injection for detail). At 1 and 4 weeks following intravitreal injections, the mice underwent simultaneous in vivo retinal imaging using a combined scanning laser ophthalmoscopy (SLO) and optical coherence tomography (OCT) imaging^16^ or electroretinography (ERG) testing. Following testing, the animals were euthanized, and the right eyes were harvested promptly for immunohistochemical studies or total RNA isolation. The detailed descriptions of each of the steps of the procedure are outlined below.

### Immunosuppression

All mice were started on systemic immunosuppression 5 days before intravitreal injection to prevent cross-species rejection of human cells.^17^ Immunosuppression was achieved by implantation of an Alzet micro-osmotic pump (model 1004; Durect Corporation, Cupertino, CA, USA), which releases immunosuppressive agents (Tacrolimus and Rapamycin) at a constant rate (1 μg/g/day each) over 1 month. Implantation was performed in all mice at 3 weeks of age.

The Alzet pumps were preloaded with FK506/rapamycin, which were diluted in 50% dimethyl sulfoxide (DMSO)/50% polyethylene glycol (PEG) and incubated in Normosol-R at 37°C for 48 hours prior to implantation. FK506 and rapamycin were purchased from InVivoGen (San Diego, CA, USA).

Prior to subcutaneous implantation of Alzet pump to the back of the mice, mice were anesthetized using isoflurane (2–3% in oxygen). Hair was removed from the back using Nair Hair Removal Lotion (Church & Dwight Co., Inc., Ewing, NJ, USA). After hair removal, the underlying skin was cleaned with betadine. After 2 minutes, the skin was wiped with an alcohol pad. A small full-thickness skin incision between the scapulae was created, which was tunneled subcutaneously toward the tail and along the back to provide a subcutaneous space large enough for the pump placement. The pump was positioned subcutaneously with the flow moderator portion of the pump pointing toward the tail and away from the incision. The skin incision was then closed with 6-0 silk sutures.

### CD34+ Cell Isolation and EGFP Labeling

Fresh human BM aspirate was purchased from AllCells (Alameda, CA, USA) and obtained for the scheduled day of use. The BM aspirate for this study was obtained from a 38-year-old healthy male donor, and CD34+ cells were isolated from the BM aspirate according to the protocol used for the clinical trial.^8^ The fresh BM aspirate was diluted 1:1 with PBS and immediately underwent Ficoll centrifugation. After centrifugation, the buffy coat was aspirated to collect the mononuclear cells. The buffy coat was diluted and washed with PBS and centrifuged to pellet all mononuclear cells. The cell pellet was then resuspended in PBS and counted using a hemacytometer. To isolate the CD34+ cells, the Miltenyi human CD34 MicroBead Kit (Cat#130-046-702; Miltenyi Biotec, Inc., San Diego, CA, USA) was used to magnetically sort out the CD34+ cells from the mononuclear cell fraction. This was done following the manufacturer's instructions.

To label the isolated CD34+ cells with enhanced green fluorescent protein (EGFP), the isolated CD34+ cells were then cultured overnight at 37°C/5% CO_2_ in a 35-mm dish in hematopoietic stem cell (HSC) Proliferation Medium as follows: Iscove's Modified Dulbecco's Medium (IMDM), penicillin/streptomycin (1×), l-glutamine (1×), 10% fetal bovine serum, stem cell factor (SCF; 50 ng/mL), thrombopoietin (TPO; 50 ng/mL), and FMS-like tyrosine kinase receptor-3 (FLT3) ligand (50 ng/mL).^18^ The following day, the cells were counted and transduced at multiplicity of infection (MOI) 20 with a lentiviral vector containing EGFP and luciferase. Briefly, the cells were pelleted and then resuspended in 50 μL HSC proliferation medium, 10 μg/mL protamine sulfate, and virus at MOI 20 in a cryo-vial. The vial was incubated for 5 hours at 37°C/5% CO_2_ and was mixed every hour to ensure maximum exposure of the cells to the virus. The cells were then resuspended in 5 mL HSC proliferation medium and replated into a 35-mm dish overnight. The following day, the cells were pelleted, washed in PBS, and then resuspended at 50,000 cells/μL in PBS for intravitreal injection.

### Intravitreal Injections

Intravitreal injections were performed 5 days after Alzet Pump implantation to allow for immunosuppression to be achieved (i.e., 4 weeks of age). An intravitreal injection was performed once in the right eye of each mouse. It was performed pars planar and transconjunctivally under isoflurane (2–3% in oxygen) anesthesia. After instilling a drop of 5% betadine solution into the fornix, a sterile 33-G needle attached to a Hamilton syringe was used to deliver 1 μL solution per eye. The animals were injected with either EGFP-labeled CD34+ cells from human BM (*n* = 16 mice, 50,000 CD34+ cells in 1 μL) or saline (*n* = 16 mice, 1 μL PBS). Following injection, antibiotic eye ointment was applied to the injected eye.

### Electroretinography

In preparation for ERG testing, the mice were dark adapted for 12 or more hours prior to testing. Pupils were fully dilated prior to testing using topical tropicamide 0.5% and phenylephrine 2.5%. For anesthesia, the animals were injected intraperitoneally with ketamine (15 μg/g) and xylazine (7 μg/g). Proparacaine 1% topical analgesic was administered to the eyes just prior to ERG electrode placement. Mice were placed on a rodent body warming plate for the duration of the procedure, and ERG was performed bilaterally. Reference needle electrodes were reconfigured into a small circular shape and bent 90° to position just over the cornea with use of goniosoft contact gel, and a reference electrode was placed subdermally between the ears towards the nose. Electrodes were held in place with use of small alligator clips. Electroretinographs were generated under a variety of conditions, including scotopic single flash at intensities of −64, −14, −8, −4, 0, and 6 dB; photopic white single flash at intensities of −64, −14, −8, −4, 0, and 6 dB; and photopic white 30-Hz flicker at 0 dB. Recordings were made using LKC Big Shot, UTAS Visual Electrodiagnostic System with EM for Windows Version 1.3 (LKC Technologies, Inc., Gaithershung, MD, USA).

### Retinal Imaging

Animals were imaged 1 or 4 weeks after intravitreal injection. A multimodal retinal imaging system specifically designed and built for in vivo mouse retinal imaging was used. This system integrates multichannel SLO and OCT and allows simultaneous collection of complementary information from the tissue, greatly simplifying data registration and analysis.^16^ With its customized scanning head, the scanning field view (FOV) can be up to 50°, whereas software control allows limiting the scanning to any square subfield of the larger field. With a customized contact lens mounted to the scan head, the mouse cornea was kept hydrated and clear, greatly facilitating mouse handling during a single imaging session.

The combined SLO and OCT imaging platform is compactly arranged in an 8 × 8-in frame and sits on a platform that can be easily tilted and translated, providing precision alignment with respect to the eye of the anesthetized mouse.

The mouse retinal imaging was performed under isoflurane (2–3% in oxygen) inhalation anesthesia. A heating pad was used to maintain normal body temperature and avoid the development of “cold cataracts” during imaging.^19^ The head was held rigidly by a “bite-bar” that also served to keep its snout inside the gaseous isoflurane anesthetic delivery tube.

#### SLO Subsystem.

A super-continuum laser (SC-400; Fianium, Inc., Eugene, OR, USA) is used as the light source for the SLO subsystem. By changing emission filters, different excitation wavelengths can be chosen. In the experiments presented here we restricted the light source spectrum to spectral band that provides strong excitation for EGFP, single bandpass filter (MF469-35; Thorlabs, Inc., Newton, NJ, USA), and chose a corresponding dichroic mirror (DM1; Di01-R488/561; Semrock, Inc., Rochester, NY, USA) and filter (FF01-525/45; Semrock) (filter 2) for EGFP emitted fluorescence light to be detected using a photomultiplier tube (PMT) (H7422-40; Hamamatsu Photonics, K.K., Shizuoka, Japan). A reflected light signal was acquired by separate PMT (H7422-20; Hamamatsu).

#### OCT Subsystem.

The Fourier domain (i.e., spectral domain) SD-OCT system imaging beam was optically integrated with the SLO subsystem via the second dichroic mirror (DM2). We used a broadband light source with a 132-nm bandwidth centered at 860 nm (Broadlighter 890; Superlum Diodes Ltd., Cork, Ireland), which provides ∼2-μm theoretical axial resolution in tissue. A custom spectrometer with a high-speed line CMOS camera (Sprint spL4096-140km; Basler Electronics, Highland, IL, USA) was used as the OCT detector. The OCT system operates at speeds up to 125,000 A-scan/s.^20^

#### Contact Lens.

A 0-diopter (D) mouse contact lens (Unicon Corporation, Osaka, Japan) was used to keep the mouse cornea hydrated. The contact lens was physically mounted on the scanning head, rather than being placed on the mouse cornea. With this approach, it is easier to keep the outer surface of the contact lens clean and, given that the mouse's head is affixed to a bite-bar embedded in the isoflurane delivery system, to maintain stable and highly reproducible imaging for periods of an hour or more if needed.

### Tissue Processing

At 1 and 4 weeks after intravitreal injections, half of the mice from each study group (i.e., *n* = 8 per study group per time point) were euthanized by asphyxiation with gaseous CO_2_ in a closed chamber following retinal imaging or ERG testing. Following euthanasia, the right eye was harvested for immunohistochemistry or microarray analysis.

For immunohistochemistry, the right eyes of four mice in each group and time point were enucleated, fixed in 10% neutral buffered formalin, and embedded in paraffin.^21^ For orientation in paraffin, the superior region of each eye was marked using tissue dye (catalog #1003-6, The Davidson Marking System; Bradley Products, Inc., Bloomington, MN, USA) before enucleating with curved scissors. The eyes were fixed by immersing in freshly prepared 10% neutral buffered formalin overnight after enucleating and subsequently embedded in paraffin maintaining orientation using the tissue dye marker. Sections were cut using a Leica RM2125RT microtome (Leica, Nussloch, Germany) at 6 μm, placed on SuperFrost Plus microscope slides, and dried overnight at room temperature. Based on the previous orientation of each eye in the paraffin embedding step, a section through the optic disk represented a sagittal section.

For microarray analysis, the right eyes of four mice in each group at each time point were removed using curved forceps and glued to a Petri dish with superglue for stabilization. A circumferential cut was made dorsal to the limbus, removing the anterior segment of the eye. Retinal tissue was removed after severing the optic nerve and collected within 1 minute after death of the animal. The collected retina was immediately placed in 300 μL RNALater, placed on ice, and stored at −20°C.

### Immunohistochemistry

Immunohistochemistry was performed by removing paraffin in xylene followed by hydration through graded alcohols and into PBS before blocking at room temperature in PBS containing 0.3% Triton X-100 and 5% normal donkey serum. Slides were then incubated overnight at 4°C with 2 μg/mL rabbit anti-GFP (Life Technologies, Carlsbad, CA, USA). After primary antibody exposure, sections were washed in PBS containing 0.3% Triton X-100 and then incubated at 37°C with 1 μg/mL Cy3 conjugated donkey anti-rabbit IgG (Jackson ImmunoResearch Labs, West Grove, PA, USA) diluted in PBS for 30 minutes, washed in PBS, and cover-slipped using 4′,6-diamidino-2-phenylindole (DAPI) Fluoromount G (Southern Biotech, Birmingham, AL, USA). Secondary antibody background staining was tested using an isotype-matched immunoglobulin (rabbit IgG; Jackson ImmunoResearch) at the same concentration as the primary antibody. Sections were also stained with anti-human nuclei monoclonal antibody (EMD Millipore, Billerica, MA, USA) to identify all injected human cells that may not have been labeled with GFP. The secondary antibody used was Alexa Fluor 647–conjugated donkey anti-mouse IgG (Millipore).

Slides were viewed and digitized images captured using a Nikon Eclipse E800 with epifluorescence and QCapture software (QImaging, Surrey, British Columbia, Canada). Digitized phase contrast with fluorescence images were captured using a Nikon Eclipse E800 microscope and QCapture software (QImaging).

### Microarray Analysis

#### RNA Isolation.

Qiagen's miRNeasy isolation kit (Valancia, CA, USA) was used to isolate total RNA. As a first step, tissue was placed in Qiazol and passed through a 1-mL syringe attached to a 17-G needle several times. RNA samples were run on an Agilent BioAnalyzer microfluidics chip RNA Nano 6000 (Agilent Technologies, Santa Clara, CA, USA) to assess quality and quantity. Of four samples, the three samples having the highest quality (RNA Integrity Number [RIN] ≥ 7 value) were labeled as probes for the Affymetrix GeneChip microarrays (Affymetrix, Santa Clara, CA, USA).

#### Microarray Probe Labeling.

Samples of RNA were labeled using 0.050 μg total RNA, following the manufacturer's protocol for GeneChip WT Terminal Labeling and Controls Kit (Affymetrix) combined with Ambion WT Expression Kit (Ambion, Inc., Foster City, CA, USA). After labeling, the probes were hybridized to the Affymetrix Mouse Transcriptome Assay 1.0 (MTA1) GeneChip microarrays. This array analyzes expression of >114,000 transcripts for protein coding genes and >101,000 transcripts for nonprotein coding content (noncoding RNA, such as microRNA, pseudogenes, and rRNA). Hybridization was performed at the University of California Davis Genome Center Microarray Core Facility using a standard manufacturer's procedure (Affymetrix). The total dataset included 12 GeneChip microarrays.

#### Microarray Data Analysis.

Microarray data were analyzed using Affymetrix Expression Console followed by Transcriptome Analysis Console 3.0 and WikiPathways for biologically relevant changes of expression in genes and related pathways of the retina. A heatmap was generated using hierarchical clustering analysis by Partek Genomics Suite software (Partek, Inc., St. Louis, MO, USA).

#### Statistical Analysis of Microarrays.

One-way ANOVA was used in the Transcriptome Analysis Console to identify statistically significant genes at a significance level of *P* ≤ 0.05. We used the standard approach of using a *P* value (*P* ≤ 0.05) as the primary criterion followed by fold change (FC) of at least 1.5 (−1.5 ≥ FC ≥ 1.5) as the secondary criterion to select differentially expressed genes. This approach ensures control of false-positive error and preserves the desired biological significance.^22^

## Results

The Table summarizes the number of mice in each study group, length of follow-up, and tests performed in this study. All mice had hereditary retinal degeneration (rd1) and were immunosuppressed systemically for the study duration. Only the right eye was injected and studied. Sixteen mice were injected with human CD34+ cells from bone marrow and 16 were injected with PBS. Half of the mice in each group were killed and analyzed 1 week following intravitreal injection, and the other half were killed 4 weeks after intravitreal injection.

**Table.** Summary of Animals, Treatments, Follow-Up, and Studies Performed in This Study

**Group**	**Mice**	**OD**	**OS**	**Follow-up**	**Studies**
CD 34+ Cell-Treated	8	CD34+	Control	1 week	Four mice underwent ERG and eyes harvested for immunohistochemistry; four mice underwent SLO-OCT imaging and retinas harvested for microarray
	8	CD34+	Control	4 weeks	Four mice underwent ERG and eyes harvested for immunohistochemistry; four mice underwent SLO-OCT imaging and retinas harvested for microarray
Control mice	8	PBS	Control	1 week	Four mice underwent ERG and eyes harvested for immunohistochemistry; four mice underwent SLO-OCT imaging and retinas harvested for microarray
	8	PBS	Control	4 weeks	Four mice underwent ERG and eyes harvested for immunohistochemistry; four mice underwent SLO-OCT imaging and retinas harvested for microarray

### In Vivo Retinal Imaging

**Figure 1:**
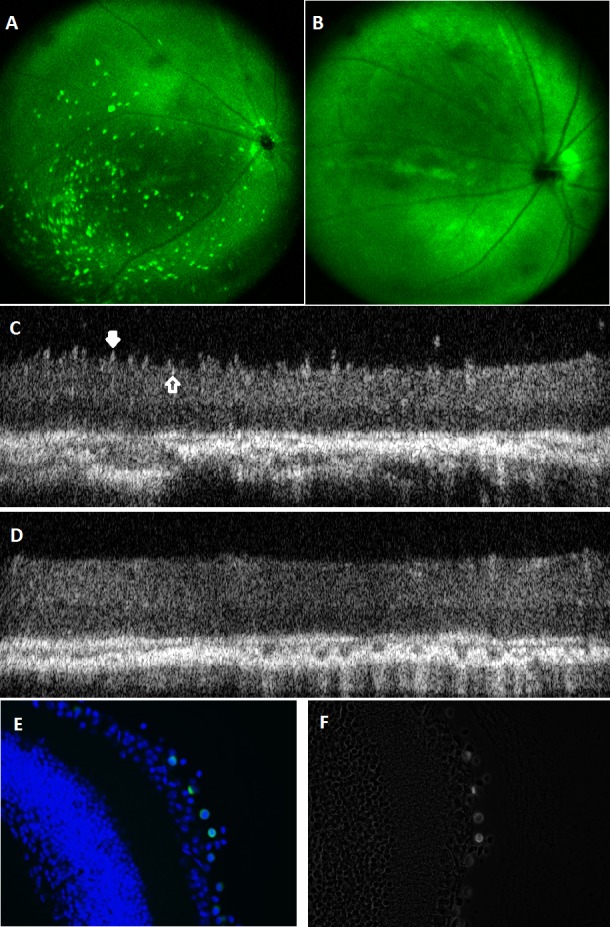
In vivo retinal imaging using combined SLO and Fourier-domain OCT retinal imaging in rd1 mice following intravitreal injection of EGFP-labeled CD34+ cells or PBS. (A) At 1 week following intravitreal injection of EGFP-labeled CD34+ cells, SLO fundus image of the right eye shows presence of EGFP-labeled cells in the eye. (B) At 1 week following intravitreal PBS injection, only background fluorescence is seen on SLO fundus image. (C) A B-scan OCT retinal image taken 1 week following intravitreal injection of EGFP-labeled CD34+ cells shows dramatic retinal surface irregularities (white solid arrow) with some focal mildly hyperreflective changes within the inner retinal layers (open arrow). These focal changes within the retina and on the surface of the retina colocalize to the fluorescent cells seen on SLO image (A). (D) A B-scan OCT retinal image taken 1 week following intravitreal injection of PBS shows a relatively smooth retinal surface without the focal hyperreflective changes within the retinal layers seen in cell-treated eyes (C). Although the outer retinal layer appears slightly more defined in this image compared with C, this was not a consistent finding. (E) Immunohistochemical analysis of the retina from rd1 mice following intravitreal injection of EGFP-labeled CD34+ cells, showing fluorescent cells on the retinal surface and adjacent to ganglion cells. (F) Phase contrast microscopy imaging of the same section shown in E, demonstrating the injected CD34+ cells on top of the retina, over the internal limiting membrane.

Combined SLO and OCT imaging of the retina of the right eye was performed on animals at 1 or 4 weeks following intravitreal injection of CD34+ cells or PBS. The CD34+ cells injected into mice eyes were labeled with EGFP to enable detection of the cells in the eye in vivo using SLO imaging. Scanning laser ophthalmoscopy fundus imaging detected the EGFP-labeled cells in eyes of mice that received these cells intravitreally. In contrast, eyes that received intravitreal PBS injection had only background fluorescence (Figs. 1A, 1B).

Simultaneous OCT and SLO imaging of the retina was conducted to obtain corresponding OCT B-scan cross-sectional images of the retina to determine the axial location of the EGFP-labeled cells seen on SLO fundus image. As shown in Figure 1C (white solid arrow), multiple focal retinal surface irregularities are noted in eyes injected with CD34+ cells resulting in a “saw-tooth” appearance at the retinal inner surface. These surface irregularities correspond in size and location to EGFP-labeled cells seen on SLO fundus photography and likely represent the presence of the EGFP-labeled CD34+ cells on the surface of the retina. In addition, Figure 1C (open arrow) also shows some mildly hyperreflective deposits seen within the inner retinal layers suggestive of intraretinal incorporation of the cells into these layers. In contrast, the OCT B-scan images of the retina of an eye injected with PBS showed a relatively smooth retinal surface (Fig. 1D). No focal hyperreflective abnormalities within the inner retinal layers are seen in the PBS-injected eyes.

At 4 weeks following intravitreal injection, a diffuse loss of the outer retina was noted on B-scan OCT images of the retina in eyes treated with either intravitreal CD34+ cells or intravitreal PBS. There was no difference in the fluorescence pattern on SLO image of eyes treated with CD34+ cells at 4 weeks compared with 1 week after cell injection. Similarly, the B-scan OCT images obtained of eyes at 4 weeks following cell injection showed similar retinal surface irregularities as was observed 1 week following CD34+ cell injection. No EGFP-labeled cells were seen on SLO fundus imaging in PBS injected eyes at 4 weeks following injection. No surface irregularities or focal intraretinal hyperreflective changes were noted on B-scan OCT images in PBS injected eyes after 4 weeks.

### Immunohistochemical Analysis

**Figure 2:**
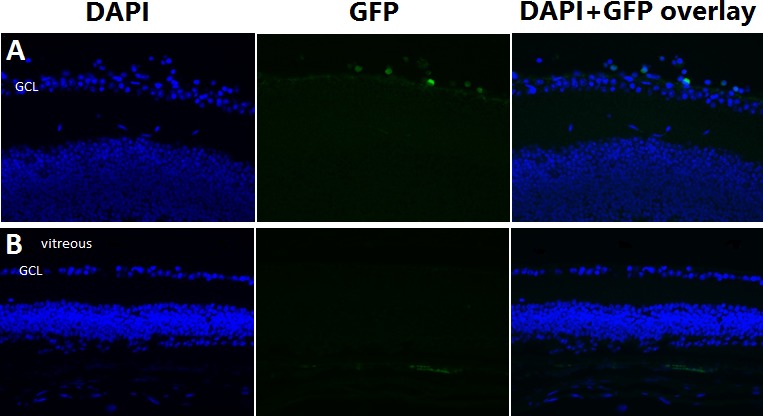
Immunohistochemical analysis of the retina from rd1 mice following intravitreal injection of EGFP-labeled CD34+ cells versus PBS. Analysis of the retina of eyes treated with intravitreal injection of EGFP-labeled CD34+ cells, the EGFP-labeled cells are detected within the vitreous and adjacent to the ganglion cell layer (GCL) at 1 week following injection (A). No EGFP-labeled cells were detected in PBS injected eyes (B). The tissue slides were blocked with 5% normal goat serum. Primary antibody used was anti-rabbit GFP. Secondary antibody used was Cy3 conjugated donkey anti-rabbit IgG.

Figure 2 illustrates the immunohistochemical analysis of eyes 1 week following intravitreal injection of EGFP-labeled CD34+ cells. EGFP-labeled cells are detected on the retinal surface and adjacent to the ganglion cells. For more detailed analysis, digitalized phase contrast microscopy imaging was used to study the histologic sections that demonstrated cells on top of the retina, adjacent to the internal limiting membrane (ILM), and ganglion cells (Figs. 1E, 1F). No EGFP-labeled cells were seen in the retina in eyes treated with PBS. Minimal background staining was seen. Similar observations were noted 4 weeks following intravitreal injection of EGF-labeled cells (data not shown; i.e., EGFP-labeled cells were detected on the retinal surface in eyes treated with CD34+ cells, similar to findings 1 week following cell injection). Additional immunohistochemical analysis of eyes treated with CD34+ cells using an anti-human nuclei antibody detected human cells on the retinal surface and adjacent to the ganglion cells, similar to findings using anti-GFP.

### Electroretinography

**Figure 3:**
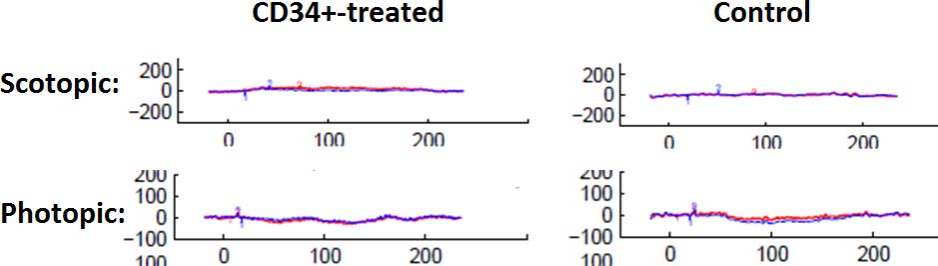
Electroretinogram of both eyes of rd1 mice at 1 week following intravitreal injection of CD34+ cells in the right eye (i.e., 4 weeks of age) under scotopic and photopic conditions. The recording is almost flat in both eyes under scotopic and photopic conditions. No difference was noted between the CD34+-treated eye (right) and untreated left eye. No difference was noted between the CD34+-treated right eye and PBS treated right eye at 1 and 4 weeks following injection. (OD, red line; OS, blue line).

The ERG testing revealed virtually flat signal recordings in all eyes under a variety of scotopic and photopic testing conditions. There were no differences in ERG testing between CD34+-injected eyes and PBS-injected eyes at either 1 or 4 weeks following intravitreal injection. There also was no difference in the ERG signal between the injected right eye and the contralateral untreated left eye (Fig. 3).

### Microarray Analysis of the RNA in the Retina

Retinas from both CD34+ cell-treated and PBS-treated control eyes were obtained at 1 and 4 weeks following intravitreal injection, and the retinal RNA was analyzed for significant changes in gene expression (i.e., FC over 1.5 and *P* < 0.05).

At 1 week following intravitreal injections, the expression of 332 mouse genes was found to be significantly changed in the retina collected from eyes treated with CD34+ cells compared with the retina from eyes treated with PBS injection. Analysis through Ingenuity Pathways Analysis (IPA) software identified that the top canonical pathway that was significantly affected was the phototransduction pathway (*P* < 0.001). IPA also performs Top Upstream Regulator analysis, which identifies a common transcription factor and a group of target genes whose expression changed. The top affected upstream regulator was CRX (*P* < 0.001), which has a known role in the development and maintenance of photoreceptors.^23^,^24^ Another significantly affected upstream regulator was NR2E3 (*P* < 0.001), which is important for photoreceptor development and has been implicated in inherited retinal dystrophies.^25^ In addition, 386 clusters of noncoding RNAs (microRNAs, small nucleolar RNAs, long noncoding RNAs) demonstrated significant changes in expression in eyes injected with CD34+ cells compared with PBS-injected eyes. The full gene lists with associated fold change of expression used for IPA analysis at 1 and 4 weeks are provided in Supplementary Tables S1 and S2.

**Figure 4:**
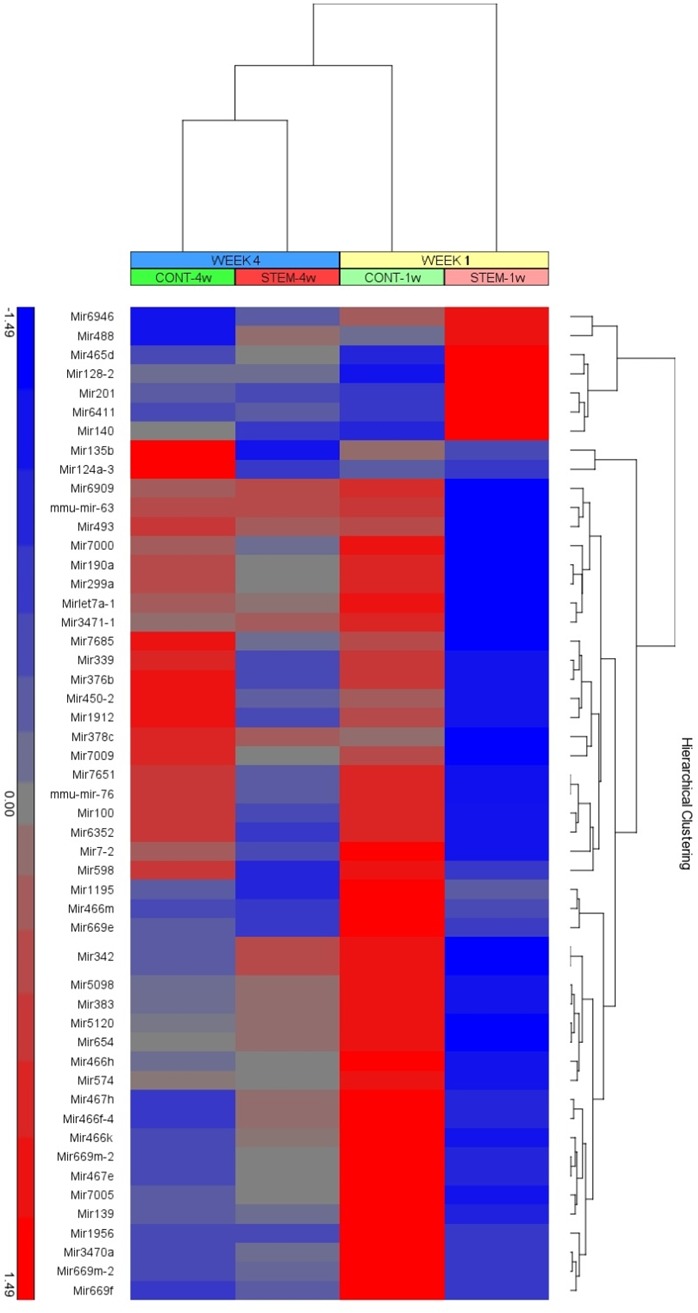
Hierarchical clustering analysis of microRNAs dysregulated (ANOVA, P < 0.05) in the retina of the mouse eyes following intravitreal injection of CD34+ stem cells and controls at 1 and 4 weeks after injection. Red and blue colors in the heatmap represent induced and repressed miRNAs, respectively. Scale bar denotes the linear values of fold change.

At 4 weeks following intravitreal injections, the expression of 86 mouse genes was found to be significantly changed in the retina of eyes treated with CD34+ cells compared with the retina from PBS-injected eyes. The top canonical pathway significantly affected at this later time point was the G-protein coupled receptor signaling pathway (*P* < 0.001). The top affected upstream regulators included PAX3, which is involved in neurogenesis,^26^ and Creb, which has been shown to have a regulatory function in apoptosis.^27^ In addition, clusters of noncoding RNAs (microRNAs, small nucleolar RNAs, long noncoding RNAs) demonstrated significant changes in expression at this time point in CD34+ cell-injected eyes compared with PBS-injected eyes. Hierarchical clustering analysis (heatmap) of microRNAs dysregulated (ANOVA, *P* < 0.05) in the retina following intravitreal injection of CD34+ stem cells and controls shows that the most dramatic differences were at week 1 and dampening at week 4 (Fig. 4).

**Figure 5:**
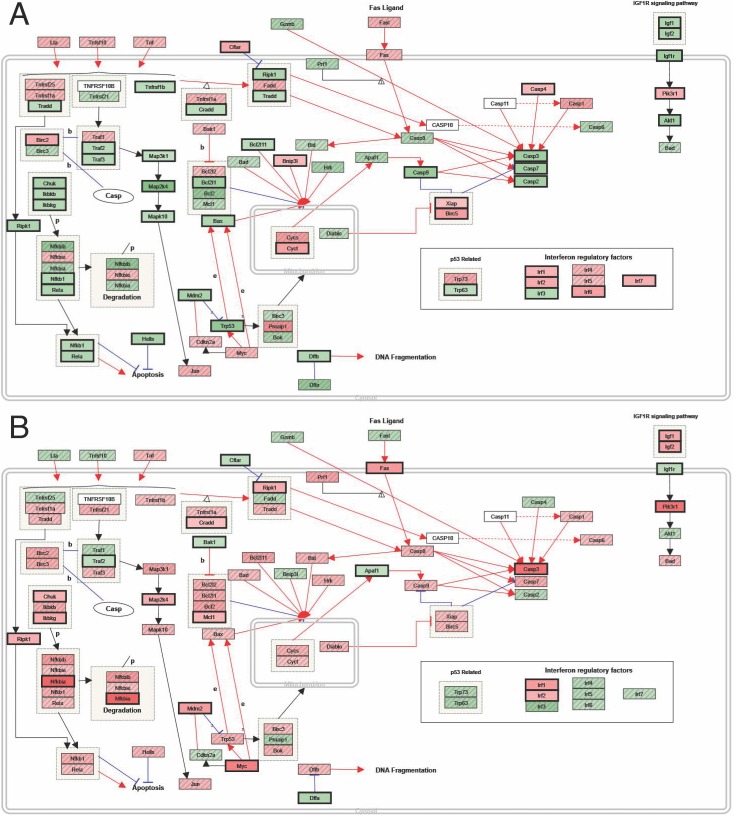
Results of microarray analysis of the RNA from the retina of rd1 mice showing the major gene expressions in the apoptosis pathway that are altered following intravitreal CD34+ cell injection compared with PBS injection. (A) At 1 week following intravitreal injection. (B) At 4 weeks following intravitreal injection. Green indicates down-regulation, and red indicates up-regulation.

A specific analysis of genes involved in the apoptosis pathway was also performed. At 1 week, a majority of these genes were down-regulated in the retina in eyes treated with CD34+ cells compared with the retina in eyes injected with PBS (Fig. 5A). At 4 weeks, this difference was diminished (Fig. 5B).

## Discussion

In this study, we used a murine model of hereditary retinal degeneration (rd1) to study the effect of human BM CD34+ stem cells on the degenerating retina following intravitreal administration. We used an in vivo retinal imaging system specifically designed for murine eyes, as well as immunohistochemical techniques to localize these cells in the eye following intravitreal administration. We then performed ERG testing and microarray analysis to determine if the intravitreal CD34+ cell treatment resulted in detectable functional or molecular changes in the retina suggestive of a therapeutic effect.

The current study was conducted to better characterize the effect of intravitreal injection of human BM CD34+ cells on the degenerating retina. This information is timely and clinically relevant because early clinical trials have been initiated exploring intravitreal administration of BM mononuclear cells or CD34+ cells as a potential therapy for various retinal degenerative conditions. Based on published results, this cell therapy appears to be feasible and without any major safety issues.^8^–^11^ Potential visual benefit was observed in some study eyes. Based on these encouraging clinical results, a larger clinical trial may be warranted to further explore the potential efficacy of this cell therapy on retinal degenerative conditions. However, before embarking on such a study, it would be important to better characterize the effects of these cells on the degenerating retina.

In our current study, the rd1 murine model was used because it is the most commonly used murine model of hereditary retinal degeneration^28^ and the mice are readily commercially available as pathogen-free animals as required for housing in a vivarium under immunosuppression. Other mouse strains with slower retinal degeneration are not readily available from vendors. However, the retinal degeneration in rd1 mice is severe and rapidly progressive, potentially making this model less ideal for detecting a therapeutic effect. A previous study exploring intravitreal autologous lineage-negative BM cell therapy used this rd1 model combined with a slower model of retinal degeneration (rd10) to show a therapeutic effect of murine lineage-negative cells in slowing retinal degeneration.^11^ The cell therapy was administered when mice were 2 weeks of age. In our study, the cell therapy was initiated at a later time point (i.e., 4 weeks of age). This is because in our study the mice had to be immunosuppressed for 5 days before initiation of human stem cell therapy and safe dosing of systemic immunosuppression in younger mice would be difficult as they experience a rapid growth phase. Because the rd1 mice typically have a flat ERG signal by 4 weeks of age, the CD34+ cell therapy was administered at an advanced stage of retinal degeneration.

Nonetheless, this murine model of rapid retinal degeneration revealed some important information about the behavior of human CD34+ cells in eyes with retinal degeneration. Although no functional differences could be detected by ERG either at 1 or 4 weeks following CD34+ cell therapy, important molecular changes were detected in the mouse retina on microarray analysis consistent with a potential trophic regenerative effect on the degenerating photoreceptors. The expression of more than 300 different mouse genes in the retina was altered by human CD34+ cell therapy within 1 week following cell administration. The most prominently affected genes were those involved in phototransduction, photoreceptor development or maintenance, and apoptosis. These molecular alterations were attenuated at 4 weeks following cell administration, although the injected cells were still detected in the eye. The attenuated effect likely reflects the advanced, perhaps near end-stage nature of the retinal degeneration at this later study time point. Retinal OCT images at 4 weeks following cell injection show a complete loss of the outer retinal layers. This diffuse loss of viable outer retinal tissue would result in attenuation of any response to cell therapy. These observations may have implications for designing an effective clinical trial to study the effects of cell therapy on progressive degenerative retinal conditions. A limited time window may be present where therapeutic effect may be observed as functional improvement.^9^

The above microarray findings are of note because there are no prior preclinical studies characterizing the molecular effect of intravitreal human CD34+ cells on the degenerating retina. Although human BM CD34+ cells are considered hematopoietic cells, the cell surface markers used to identify these human hematopoietic cells are different from cell surface markers used to identify murine hematopoietic cells, and murine CD34+ cells behave differently from human CD34+ cells.^9^ Human CD34+ cells have been used successfully for BM transplantation in patients with hematologic disorders. However, these CD34+ cells are thought to consist of a heterogeneous group of cells. Currently, human CD34+ cells are being explored as a potential regenerative therapy for ischemic conditions, such as ischemic cardiomyopathy or peripheral ischemia, because this cell population includes endothelial progenitor cells.^13^

There is no published information on the potential neuro-protective effects of CD34+ cells on the degenerating retina. However, these cells appear to be recruited from the BM to the degenerating retina and retinal pigment epithelium. An elevated level of CD34+ cells in the circulation has been noted in patients with active exudative AMD.^29^ In addition, in an animal model of retinal pigment epithelial degeneration, BM CD34+ stem cells have been detected to be recruited to the site of degeneration.^30^

In this study, CD34+ cells were isolated from the mononuclear cellular fraction of the BM aspirate using a magnetic cell sorter, similar to the method used for the ongoing clinical study.^8^ The number of CD34+ cells injected intravitreally in mice is similar to the number of cells that have been injected in human eyes as part of the clinical trial when adjusted for differences in vitreous volume between species. The isolated CD34+ cells were labeled with EGFP prior to intravitreal injection to follow the migration of these cells within the eye using in vivo retinal imaging and immunohistochemistry.

The study results show evidence that intravitreally injected CD34+ cells from human BM can migrate to the retinal surface and become tightly associated with the retina. Although transretinal migration of CD34+ cells was not detected in this study, some intraretinal hyperreflective changes were noted on OCT, resembling those observed with the in vivo cellular retinal imaging using adaptive optics OCT (AO-OCT) imaging of the retina in a patient with hereditary macular degeneration in the clinical trial.^8^ These in vivo imaging changes within the retina are likely related to the presence of the CD34+ cells.

The results of in vivo SLO-OCT retinal imaging conducted in murine eyes in this study show some important differences compared with the AO-OCT images on the human subject. Because of the smaller size of the murine eye and relatively large pupil compared with the human eye (which result in a higher numerical aperture), as well as the application of a contact lens, the combined in vivo SLO and OCT retinal imaging could be used to achieve near cellular axial and lateral resolution of the images of the retina without incorporating AO.^31^ Perhaps because of these differences in imaging techniques, the intraretinal reflective changes noted in the murine retina following CD34+ cell therapy were more subtle that those noted in the human study. In fact, the majority of the injected CD34+ cells appear to be attached to the retinal surface in a diffuse sheet-like manner, creating a dramatic saw-tooth appearance to the retina surface on OCT B-scan imaging of the murine retina. No such retinal surface changes were noted in the human subjects using OCT or AO-OCT imaging.^8^

There are several possible explanations for this observed difference. First, it is possible that the vitreo-retinal interface in these murine eyes with hereditary retinal degeneration might differ from that in human eyes with hereditary retinal degeneration, and the difference might affect the chemotactic signal or migration behavior of these cells. Some species variation in retinal penetration of intravitreally injected proteins or drugs have been observed.^32^,^33^ Second, the possible effect of concurrent systemic immunosuppression on the migration and behavior of the CD34+ cells cannot be ruled out. Previous cross-species studies using human CD34+ cells in mice have not used as extensive systemic immunosuppression as was used in this study, because these prior studies were short-term studies.^33^,^34^ These earlier studies have shown retinal vascular incorporation and transretinal migration of intravitreally injected human CD34+ cells in animal models of diabetic retinopathy or laser retinal injury. Last, the less dramatic intraretinal incorporation of the injected cells seen in this current study could represent some loss of viability of these cells following isolation and overnight incubation of the CD34+ cells for EGFP labeling. However, this is unlikely because long-term intraretinal viability of these cells has been demonstrated in previous works using NOD-SCID mice,^15^ and the extent of detection of EGFP-labeled CD34+ cells in the retina was unchanged at 1 and 4 weeks after injection in our current study. In addition, previous studies have shown that human CD34+ cells can be stably transduced by lentiviral vectors.^18^ Nonetheless, the injected CD34+ cells likely secrete factors such as cytokines and microRNAs that traverse the retina and induce changes in the retinal cells. This paracrine effect would explain the molecular changes within the mouse retina noted in this study following CD34+ cell injection, even in the absence of detectable migration of CD34+ cells through the retina to the degenerating outer retina.

The potential therapeutic effect of the intravitreally injected CD34+ cells on the degenerating retina is evident based on the dramatic molecular changes within the mouse retina detected using microarray analysis despite the relatively advanced stage of murine retinal degeneration at the time of cell therapy. Because these molecular changes in the mouse retina were noted in the absence of any detectable direct incorporation of the CD34+ cells into the degenerating photoreceptor layer of the retina, a paracrine trophic effect is implicated. The findings are analogous to results of a previous study using intravitreal autologous lineage negative hematopoietic bone marrow cells in mice. The injected murine cells were localized to the retinal vasculature, although some preservation of the cones and down-regulation of apoptotic genes were noted.^11^

CD34+ cells are an attractive candidate for stem cell therapy, as they can be obtained relatively easily from patients, require minimal manipulation prior to administration, and can be used in an autologous procedure, thus obviating the need for finding a compatible donor or for immunosuppression. Our previous work has established that they are capable of integrating into the retinal vasculature in NOD-SCID mice with acute ischemia-reperfusion injury following intravitreal injection, and remain there long term with no ocular and systemic adverse effects.^15^ In our current study, intravitreal injection of human BM CD34+ cells was well tolerated in eyes with hereditary retinal degeneration with no ocular or systemic adverse effects noted during the course of the study. Results indicate that the cells were able to survive in murine eyes for at least 4 weeks. Even though no changes in retinal function could be demonstrated by ERG in these eyes with advanced retinal degeneration, the gene expression changes in the degenerating retina were demonstrated by microarray analysis in eyes treated with CD34+ cells. Alterations in the expression of genes for proteins regulating photoreceptor preservation and apoptosis suggest a potential therapeutic effect on retinal degeneration that warrants further investigation. In addition, alterations in noncoding RNAs associated with CD34+ cell injection further support a potential therapeutic effect of this cell therapy because noncoding RNAs are known to have key roles in gene expression regulation at the posttranscriptional level.

Our study had several limitations. First, systemic immunosuppression was used to allow human cell engraftment in transgenic, immune competent mice, which may have influenced the behavior of the injected CD34+ cells and the degenerating retina. Second, although EGFP labeled the majority of the isolated CD34+ cells, the labeling was not complete and may not have been strong enough to detect all of the CD34+ cells in the eyes. Because the isolation of CD34+ cells from BM yields a relatively small amount of these cells, flow cytometry was not performed on the CD34+ cells following EGFP labeling to determine the percentage of cells labeled with EGFP after transduction. Nonetheless, previous studies have demonstrated that this method of transduction using a lentiviral vector achieves a relatively high rate of transduction in CD34+ cells.^18^ Additional immunohistochemical analysis using anti-human nuclei antibody revealed results similar to studies using anti-EGFP antibody (i.e., no human cells were identified beyond the superficial layers of the retina). Although incorporation of the cells into the outer retina could not be clearly demonstrated by immunohistochemistry, this possibility cannot be ruled out. Transretinal migration of CD34+ cells following intravitreal injection has been demonstrated previously using a retina laser injury model.^34^ Third, treating the mice at almost 4 weeks of age likely attenuated the potential therapeutic effects of the CD34+ cells on the degenerating retina. The rd1 retinal degeneration is very rapid and severe, and it is possible that a more robust therapeutic effect might be achieved in murine models with slower rate of retinal degeneration, such as rd10 or rd19. These mice with slower retinal degeneration were not readily available for the current study. Despite these limitations, the findings of this study support further investigation of the therapeutic potential of CD34+ cells on the degenerating retina.

In conclusion, this study sheds new light on the behavior of human CD34+ cells following intravitreal injection in murine eyes with retinal degeneration. The dramatic chemotactic response of the intravitreally injected CD34+ cells to the degenerating retina is demonstrated using in vivo retinal imaging and immunohistochemistry by the diffuse, tight adherence of the cells to the retinal surface. The dramatic molecular changes induced within the degenerating retina following this cell therapy support a potential therapeutic effect that warrants further investigation of intravitreal CD34+ cell therapy as a potential treatment for retinal degenerative conditions.

## Supplementary Material

Supplement 1

Supplement 2
